# Awake Forceps Retrieval of Complex Pharyngeal Fish Bone Impactions Using a Single-Use Nasendoscope With an Instrument Channel: A Retrospective Case Series

**DOI:** 10.7759/cureus.101332

**Published:** 2026-01-12

**Authors:** Ian T Braithwaite, Max B Butler, Dave K Sharma, Oloruntobi Rotimi, Isabelle Wood, Claudia Nogueira

**Affiliations:** 1 Otolaryngology, Royal London Hospital, Barts Health NHS Trust, London, GBR

**Keywords:** airway foreign body, ent emergency, fish bone, fish bone ingestion, nasendoscopy, pharyngeal foreign body

## Abstract

Objective: Pharyngeal fish bone impaction is a common ENT emergency with significant potential morbidity. Most cases are managed perorally, but deeper or complex impactions often require removal under general anaesthesia (GA). Retrieval using forceps through the working channel of a nasendoscope is an established alternative, though uptake has been limited by the decontamination requirements of reusable channelled nasendoscopes. Single-use channelled nasendoscopes may enable wider adoption of this technique, improving care and reducing costs. This study aims to evaluate the clinical effectiveness and economic impact of awake single-use channelled nasendoscope-guided retrieval of pharyngeal fish bones compared with removal under GA.

Methods: A retrospective case series at a tertiary London hospital reviewed adults treated between February and May 2025 for pharyngeal fish bone impaction unsuitable for peroral extraction. Awake retrieval was performed using a single-use channelled nasendoscope and flexible crocodile forceps. Data were analysed for success, complications, GA requirement, and costs using NHS England 2024/25 tariffs and device prices.

Results: A total of 10 patients (median age 53.5 years, range 37 to 80 years; 7:3 female:male) met the inclusion criteria. Nasendoscopic retrieval succeeded in 8/10 cases (80%) with no complications. Two patients required theatre removal under GA. The mean cost per nasendoscopic case was £202 compared with £3,922 for GA removal, saving £3,720 per successful case (94.8% reduction). Cost modelling showed the technique remained cost-neutral above a 5.2% success rate.

Conclusion: Awake single-use channelled nasendoscope-guided removal of pharyngeal fish bones is safe, effective, and substantially cheaper than GA removal. Wider adoption could reduce theatre demand, anaesthetic exposure, and overall healthcare costs.

## Introduction

Pharyngeal fish bone impaction is a common emergency presentation encountered in otolaryngology. Fish bones are the commonest foreign body in the upper aerodigestive tract, accounting for up to 88% of impactions [1]. Their incidence is highest among Asian, Mediterranean, and coastal populations secondary to high dietary fish consumption [2].

Impacted fish bones carry a recognised risk of significant morbidity, including airway compromise, mucosal perforation, abscess formation, and migration [3]. Rare but life-threatening complications, such as carotid artery injury [4], retropharyngeal abscess, cervical spinal epidural abscess [5], and mediastinitis, have also been reported. Prompt removal is therefore essential, with the approach determined by the anatomical site of impaction. The most frequent sites, in descending order of frequency, are the tonsils, base of tongue, vallecula, and pyriform sinus [6].

Foreign bodies at the tonsil or tongue base are generally amenable to peroral retrieval under direct vision using forceps with topical anaesthesia. In contrast, impactions within the vallecula or pyriform fossa are technically more challenging. Contributing factors include the size and orientation of the fish bone, anatomical variation, craniofacial abnormalities, lingual tonsillar hypertrophy, and the presence of a hyperactive gag reflex despite adequate anaesthesia. Patient comorbidities, particularly those limiting cervical spine mobility, advanced age, operator experience, and patient compliance, may further compound difficulty.

For these more complex cases, awake removal may be attempted under direct laryngoscopy using Magill forceps or with the aid of a flexible nasendoscope while a second operator manipulates forceps perorally. These techniques depend on a high degree of patient cooperation and are subject to the same limiting factors outlined above. When awake retrieval is unsuccessful or deemed unlikely to succeed, patients require emergency theatre for direct laryngoscopy/panendoscopy under general anaesthesia (GA). This introduces treatment delay, exposes the patient to anaesthetic and procedural risks, and imposes substantial additional cost and resource burden on ENT and emergency theatre services.

Retrieval using crocodile forceps through the working channel of a nasendoscope is an established technique for pharyngeal fish bone retrieval [7-9]. However, uptake in the United Kingdom has been restricted by the cost and decontamination requirements of reusable channelled nasendoscopes, in line with NHS guidance on endoscope sterilisation [10]. As a result, many ENT services lack access to this equipment for emergency use in fish bone removal.

The introduction of single-use fibre-optic nasendoscopes with a working channel has the potential to broaden the adoption of this technique. By obviating the need for costly decontamination and reprocessing while maintaining the functionality of reusable channelled scopes, single-use devices may enable wider adoption and improved patient outcomes while simultaneously offering potential cost savings. This study retrospectively evaluates the clinical effectiveness and economic impact of awake single-use channelled nasendoscope-guided retrieval of pharyngeal fish bones not amenable to peroral removal, compared with removal under GA in theatre.

## Materials and methods

Objective and methods

This study aimed to evaluate the clinical effectiveness and economic impact of awake single-use channelled nasendoscope-guided retrieval of pharyngeal fish bones not amenable to standard per-oral techniques, compared with removal under general anaesthesia in theatre. A retrospective case series was conducted at a tertiary London hospital, reviewing consecutive adult patients (>18 years) who presented between February and May 2025 with pharyngeal fish bone impaction not amenable to standard peroral retrieval.

Inclusion and exclusion criteria

Adult patients with a visualised pharyngeal fish bone unsuitable for conventional peroral extraction in whom nasendoscope-guided forceps retrieval was attempted and who, in the absence of this technique, would have required emergency theatre for removal under GA were included in the study. Patients with oesophageal foreign bodies were excluded.

Procedure

**Figure 1:**
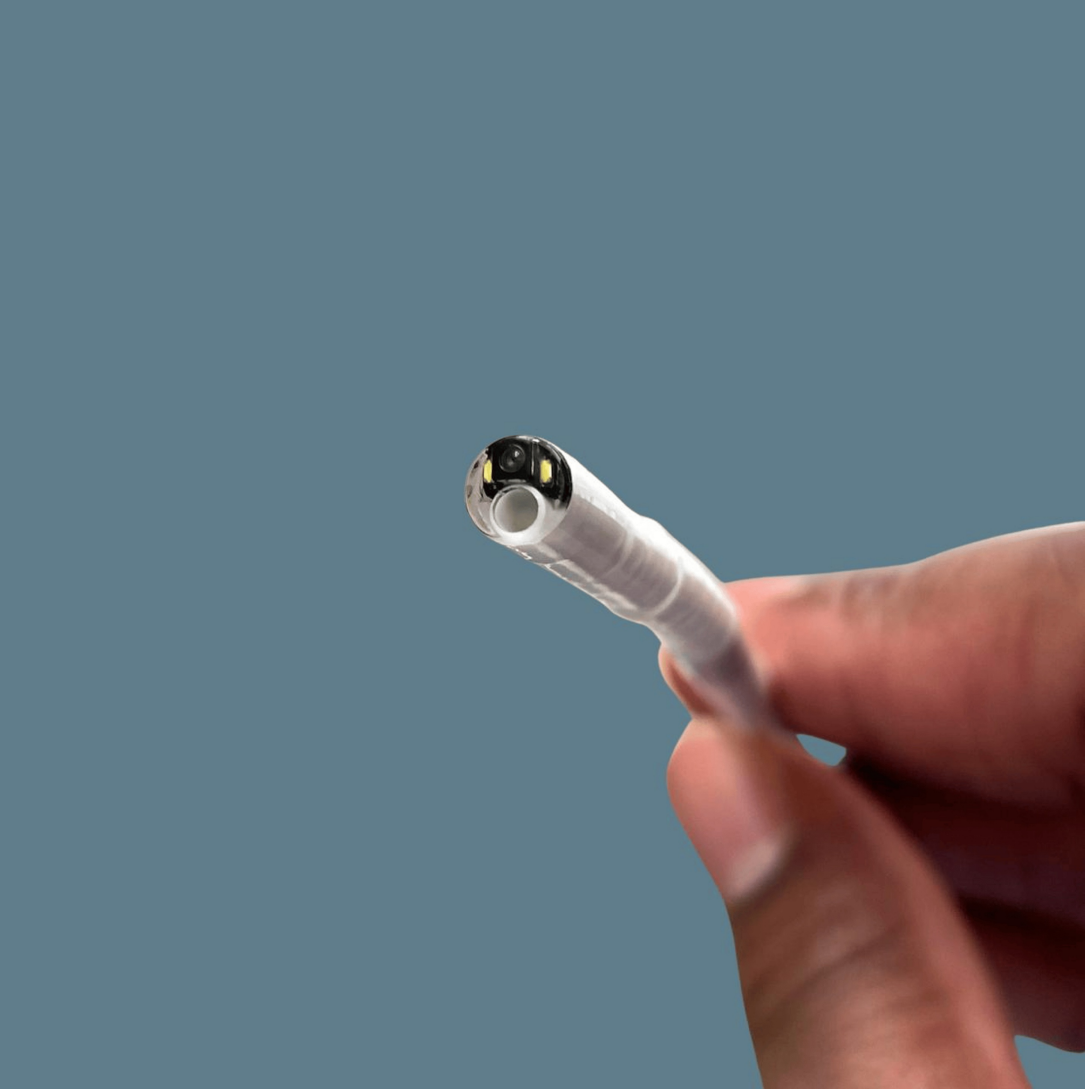
Distal end of single-use channelled nasendoscope showing camera and working channel Photo captured by author Braithwaite.

**Figure 2:**
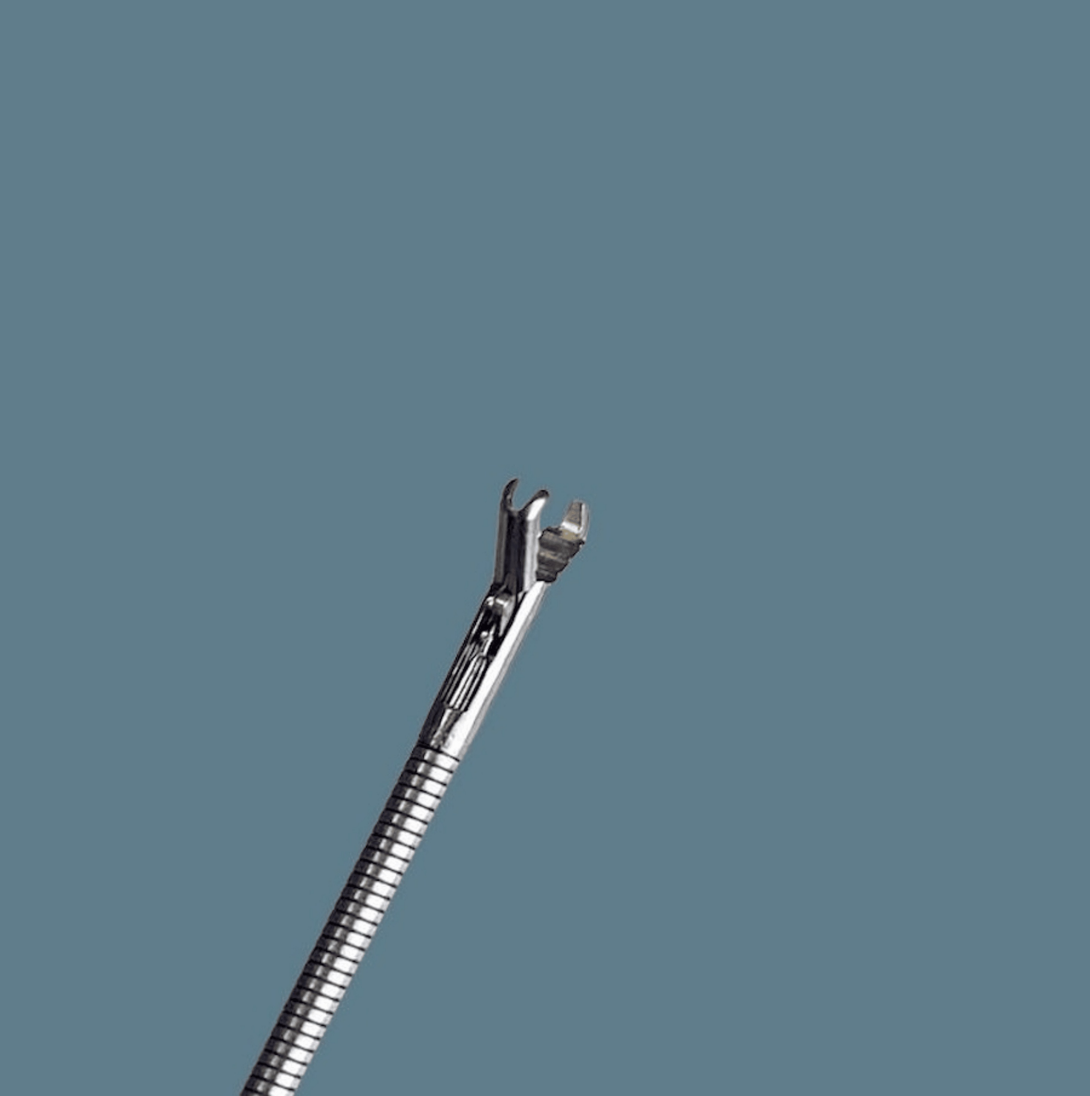
Flexible crocodile forceps in open position Photo captured by author Braithwaite.

**Figure 3:**
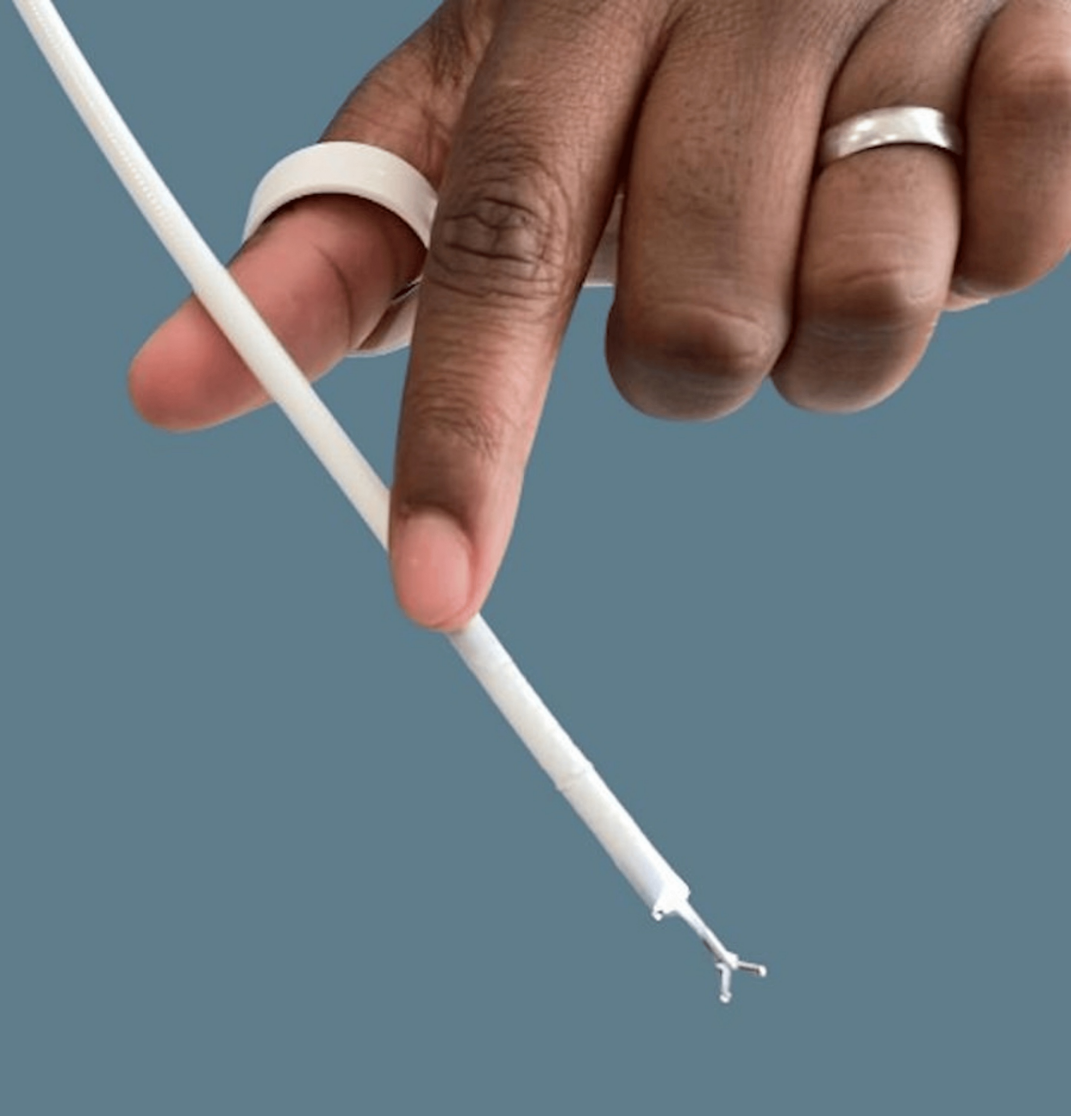
Single-use channeled nasendoscope with crocodile forceps passed through the working channel Photo captured by author Braithwaite.

Awake retrieval under topical anaesthesia was performed using a single-use channelled nasendoscope with a working channel for instruments, through which flexible crocodile forceps were passed. All procedures were undertaken as part of standard clinical care by on-call otolaryngology trainees trained in endoscopic pharyngeal foreign body retrieval. The setup and components of the single-use channelled nasendoscope system and forceps are shown in Figures 1-3.

Data collection and analysis

Data was extracted from clinical records and included demographics, anatomical site of impaction, retrieval success, need for assistance from a second otolaryngologist, complications, requirement for theatre removal under GA, and hospital admission. Economic evaluation was performed using the NHS England 2024/25 reference costs and published equipment prices. The tariff for a non-elective 'CA68 therapeutic endoscopic larynx or pharynx procedure' (inclusive of hospital admission) was £3,922 [11]. The procurement cost of a single-use flexible nasendoscope and crocodile forceps was £202 [12]. The cost of the reusable video monitor was excluded, and ENT staff costs were not incorporated on the basis that the on-call otolaryngology team was equivalent across both pathways, with theatre staff included within the quoted NHS procedural cost. Net savings per case were calculated, and cost-benefit analysis determined the breakeven retrieval success rate at which the nasendoscopic technique remained cost-neutral.

## Results

A total of 10 patients met the inclusion criteria (median age 53.5 years, range 37 to 80 years; 7:3 female:male). Two patients with oesophageal impaction were excluded. The vallecula was the most frequent site of impaction (n=4), followed by the tongue base (n=3), pyriform fossa (n=2) and posterior tonsillar pillar (n=1). Awake retrieval using the single-use nasendoscope was successful in eight of 10 cases (80%). Two patients required subsequent escalation to theatre. A second otolaryngologist was required to assist in three cases (30%). No complications were observed, including epistaxis, mucosal perforation, bleeding, airway compromise, or infection.

The mean cost per nasendoscope case was £202, compared with £3,922 for theatre removal under GA. The mean saving per successful case was £3,720 (94.8% reduction). Cost-benefit analysis demonstrated that attempting nasendoscopic retrieval remained economically advantageous above a success threshold of 5.2%, substantially lower than the observed successful retrieval rate of 80%.

**Figure 4:**
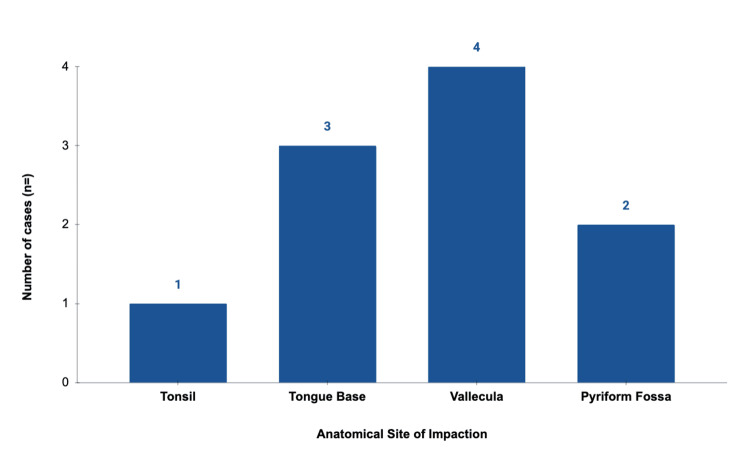
Anatomical sites of fish bone impaction

**Figure 5:**
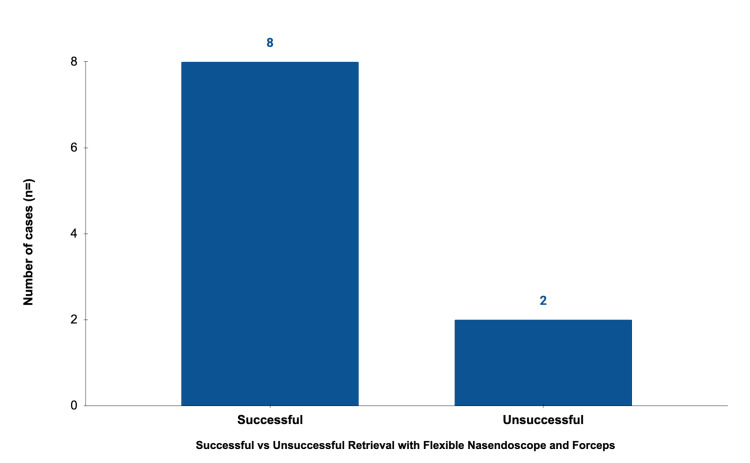
Retrieval success rates for awake single-use nasendoscopic removal

Illustrative data and visual summaries are presented below. The anatomical distribution of impaction sites is shown in Figure 4, and retrieval success rates in Figure 5. Individual case characteristics, including site, outcome, and requirement for GA, are detailed in Table 1. A representative video demonstrating successful awake retrieval from the right pyriform fossa is provided as supplementary Video 1.

**Table 1:** Patient demographics, anatomical site of impaction, need for admission, retrieval outcome (including need for a second operator to assist awake retrieval), and requirement for escalation to GA GA: General anaesthesia

Age	Sex	Site of impaction	Laterality	Admitted	Successful retrieval	Second operator	Emergency theatre
37	Female	Tonsil (posterior tonsillar pillar)	Left	Yes	No	No	Yes
65	Male	Tongue base	Right	No	Yes	No	No
51	Female	Tongue base	Right	Yes	Yes	Yes	No
58	Female	Tongue base	Right	No	Yes	No	No
60	Female	Vallecula	Right	No	Yes	No	No
51	Male	Vallecula	Right	No	Yes	Yes	No
55	Male	Vallecula	Right	No	Yes	No	No
52	Female	Vallecula (submucosal)	Right	Yes	No	No	Yes
37	Female	Pyriform fossa	Left	Yes	Yes	Yes	No
80	Female	Pyriform fossa	Right	No	Yes	No	No

**Video 1:** Successful awake nasendoscopic retrieval of a fish bone embedded in the right pyriform fossa Video captured by author Braithwaite.

## Discussion

Fish bones located within the oral cavity or tonsillar region are generally amenable to peroral retrieval under direct vision using forceps [13]. In contrast, fish bones impacted deeper within the oropharynx and hypopharynx are recognised to be more technically challenging to remove and are associated with higher rates of failed peroral extraction and escalation to removal under GA [14]. These more complex impactions formed the exclusive focus of this case series.

A wide range of awake techniques have been described in the literature to manage these impactions, including direct laryngoscopy with Magill forceps [15], video laryngoscopy with forceps [14,16-18], flexible nasendoscopy with peroral forceps (single- or dual-proceduralist) [19,20], per-oral rigid endoscopy with forceps [21], and flexible nasendoscopy with a flexible suction catheter passed via the contralateral nasal cavity [22]. There is consensus across the literature that fish bones impacted within the upper aerodigestive tract should be removed as promptly as possible, while ensuring patient safety, to reduce the risk of significant potential complications [3].

In this case series, we observed an 80% successful retrieval rate using flexible nasendoscopy with flexible crocodile forceps passed through the instrumentation channel. This is broadly in keeping with the published literature, although direct comparison is limited by heterogeneity between studies and a relative paucity of published data. In an early series of 178 pharyngeal fish bone impactions (not limited by complexity or anatomical sub-site), 73% were removed perorally, 15% were removed using nasendoscopy with forceps, and 12% required escalation to theatre for fish bones at or below the level of the cricopharyngeus [7]. More recent small case series [23] and case reports [9,24] have also described high success rates.

The high, successful retrieval rate observed in this series, and across the literature, likely reflects the mechanical and ergonomic advantages of nasendoscopy. Stable endoscopic visualisation combined with fine instrument control allows atraumatic manipulation within pharyngeal spaces without stimulating a gag reflex. This is particularly relevant for impactions within the vallecula and pyriform fossa, where limited space necessitates precise retrieval around laryngeal structures.

To our knowledge, this is the first case series to evaluate the use of single-use channelled nasendoscopes for awake pharyngeal fish bone removal and the first to formally assess the associated economic impact. This is of particular relevance given the lack of availability of reusable channelled nasendoscopes to on-call ENT services due to complex decontamination requirements. While previous studies have suggested that awake nasendoscopic techniques can reduce theatre utilisation and anaesthetic exposure [7-9,13], these assertions have largely been qualitative. In the present series, formal cost modelling demonstrated a mean cost of £202 per awake nasendoscopic case compared with £3,922 for removal under GA, representing a 94.8% reduction in procedural cost. Cost-benefit analysis showed that the technique remained economically advantageous above a retrieval success rate threshold of just 5.2%. From an economic perspective, the observed cost-benefit was substantial. The breakeven threshold for successful retrieval of approximately 5% indicates a robust margin of cost-effectiveness even with variability in operator experience or success rate. At a system level, adopting awake nasendoscopic retrieval could reduce emergency theatre utilisation, shorten time to definitive management, and improve emergency department flow, which are benefits of particular relevance in the context of current healthcare service pressures.

The absence of complications in this series is notable, particularly given that no cases of epistaxis were observed despite the passage of the scope, retrieval instrument, and retrieved foreign body via the nasopharynx and nasal cavity. This likely reflects the pliable nature of fish bones, which tend to deflect away from the direction of withdrawal, thereby minimising mucosal trauma during extraction. The two cases requiring conversion to GA do illustrate limitations of the technique: the first involved a submucosal impaction that ultimately remained irretrievable even in theatre. The second was impacted within the posterior tonsillar pillar and deflected laterally, preventing adequate instrument angulation and working space. This is consistent with previous studies on complex pharyngeal fish bone impactions, which identified posterior tonsillar fish bones as the most complex of tonsillar impaction sites [14].

This technique also lends itself to structured training and simulation. Prior studies have demonstrated that operator experience has a marked impact on patient outcomes [25]. Awake channelled nasendoscopic retrieval is well suited to simulation-based training using simple models, allowing trainees to develop competence in visualisation, instrument handling, and coordination in a controlled environment. Incorporation into defined management pathways for pharyngeal fish bone impaction could formalise patient selection, escalation criteria, and competency frameworks, supporting consistent, safe implementation across on-call ENT services.

Limitations of this study include its retrospective design, small sample size, and single-centre setting. Operator discretion in determining suitability for awake retrieval introduces potential selection bias, and outcomes may vary with experience. Future multicentre studies should prospectively evaluate patient tolerability, training requirements, and pathway-level outcomes to better define the role of awake nasendoscopic retrieval in managing pharyngeal fish bone impaction. Nevertheless, the findings demonstrate that awake single-use channelled nasendoscope-guided retrieval is a safe, effective, and economically favourable addition to established management strategies for complex pharyngeal fish bone impaction.

## Conclusions

Pharyngeal fish bone impactions are a common ENT presentation with significant potential morbidity. While the majority can be removed simply, complex pharyngeal impactions often necessitate escalation to emergency theatre with associated risks and costs. Awake forceps retrieval of complex pharyngeal fish bone impactions using a single-use channelled nasendoscope is a safe and effective alternative to removal under GA. In this series, the technique demonstrated a high success rate with no observed complications and was associated with substantial cost savings. With the broader availability of single-use instruments, greater uptake of this technique has the potential to improve patient outcomes and reduce costs.
